# Potential causes of sudden cardiac death in nemaline myopathy

**DOI:** 10.1186/s13052-015-0175-x

**Published:** 2015-09-29

**Authors:** Josef Finsterer, Marlies Frank

**Affiliations:** Krankenanstalt Rudolfstiftung, Postfach 20, 1180 Vienna, Austria; 1st Medical Department, Krankenanstalt Rudolfstiftung, Vienna, Austria

**Keywords:** Non-compaction, Cardiomyopathy, Myopathy, Cardiac involvement, Sudden cardiac death, Ventricular arrhyhtmias

## Abstract

ᅟ

## Letter to the Editor

With interest we read the article by Marseglia *et al.* about a 6yo boy with nemaline myopathy (NM) but without evidence for previous cardiac disease who experienced non-triggered asystole, underwent prolonged resuscitation, and died 1 week later from secondary cerebral hypoxia [1]. We have the following comments and concerns.

Was the previous history positive for palpitations, collapse, syncope, exertional dyspnea, or leg edema? Cardiac involvement in NM has been previously reported and includes sino-atrial block [2], WPW-syndrome [2], atrial fibrillation [3], bundle branch block [4], left anterior hemiblock [4], hypertrophic cardiomyopathy [5], outflow tract obstruction [6], dilative cardiomyopathy [5], pulmonary hypertension [4], heart failure [7], and sudden cardiac death [5] (Table 1). Did the patient undergo long-term ECG recording with 24 h-ECG, telemetry, or a reveal recorder?

**Table 1:** Manifestations of cardiac involvement in nemaline myopathy

Cardiac abnormality	NOP	Sex	Age (y)	Reference
SA-block	1	nm	nm	[2]
WPW-syndrome	1	nm	nm	[2]
Atrial fibrillation	1	m	69	[3]
Incomplete BBB	1	m	50	[4]
Complete BBB	1	m	47	[10]
Left anterior hemiblock	1	m	50	[4]
Pulmonary hypertension	1	m	50	[4]
dCMP	6	f, 4 m, nm	9, 26, 29, 47, 50, nm	[4, 5, 11–13]
hCMP	8	5 m/2 nm	20/0/2/4/5/0/nm	[6, 7, 10, 14–19]
Outflow tract obstruction	1	nm	Neonate	[6, 14]
Heart failure	4	f, 2 m, nm	0/9, 3, nm	[5, 7, 20]
Sudden cardiac death	2	f	47, 37	[5]

*NOP* Number of patients, *SA* Sino-atrial, *WPW* Wolff-Parkinson-White, *BBB* Bundle branch block, *dCMP* Dilated cardiomyopathy, *hCMP* Hypertrophic cardiomyopathy, *nm* Not mentioned

Did laboratory parameters on admission, such as serum potassium, creatine-kinase, troponine-T, or proBNP, indicate cardiac disease? Was there any indication for intoxication or metabolic defect on blood gas analysis? Was there any indication for infectious disease, such as increased C-reactive protein, increased procalcitonin, or leukocytosis? Did the patient receive cardiotoxic medication prior to the event?

Were there indications on ECG or echocardiography that sudden cardiac death resulted from stress cardiomyopathy, also known as Takotsubo cardiomyopathy, broken heart syndrome, or stunned myocardium? Takotsubo syndrome may occur even in the absence of systolic dysfunction [8]. Was there any possible trigger leviable shortly before the event that could have induced anxiety? Was the patient exposed to physical or psychological stress prior to the event?

Were previous echocardiographies reviewed for left ventricular hypertrabeculation, also known as noncompaction (LVHT)? LVHT is frequently associated with embolism, heart failure, ventricular arrhythmias, and sudden cardiac death. LVHT is frequently missed on transthoracic echocardiography if the apex is not well depicted. Was there any indication for LVHT on the last bed-side echocardiography before decease? Did the patient undergo cardiac MRI? Did the parents undergo echocardiography to look for subclinical cardiac involvement?

Did the pathologist look for fibrosis of the cardiac conduction system? Was there any indication for endocardial fibrosis on myocardial autopsy frequently found in patients with LVHT [9]. Was there histological evidence for myocarditis? Why did the heart and cerebrum not undergo autopsy? LVHT can be easily missed on echocardiography and cardiac MRI? Autopsy is the golden standard to confirm LVHT. Was cerebral imaging carried out prior to the event? Embolic stroke may secondarily cause arrhythmias, particularly if the temporal lobe is affected from ischemia.

The most frequent of the cardiac abnormalities in NM is hypertrophic cardiomyopathy, followed by dilated cardiomyopathy and heart failure (Table 1). Only in 2 patients with NM has been sudden cardiac death so far reported (Table 1). Various different ECG abnormalities have been reported only in single patients (Table 1).

Overall, this interesting case would have deserved more and thorough work-up for the cause of cardiac conduction disturbance. It is also essential to screen the whole family not only for NM but also for cardiac involvement, in particular ventricular arrhythmias and LVHT. Sudden cardiac death in the presented patient may not only be attributed to arrhythmias but also to embolic stroke, TTS or LVHT.

## References

[1] Marseglia L, D’Angelo G, Manti S, Salpietro V, Arrigo T, Cavallari V, Gitto E (2015). Sudden cardiac arrest in a child with nemaline myopathy. Ital J Pediatr.

[2] Otsuji Y, Osame M, Tei C, Minagoe S, Kisanuki A, Arikawa K, Saito K, Nomoto K, Kashima T, Tanaka H (1985). Cardiac involvement in congenital myopathy. Int J Cardiol.

[3] Jones JG, Factor SM (1985). Familial congestive cardiomyopathy with nemaline rods in heart and skeletal muscle. Virchows Arch A Pathol Anat Histopathol.

[4] Taglia A, D’Ambrosio P, Palladino A, Politano L (2012). On a case of respiratory failure due to diaphragmatic paralysis and dilated cardiomyopathy in a patient with nemaline myopathy. Acta Myol.

[5] Meier C, Voellmy W, Gertsch M, Zimmermann A, Geissbühler J (1984). Nemaline myopathy appearing in adults as cardiomyopathy. A clinicopathologic study. Arch Neurol.

[6] Mir A, Lemler M, Ramaciotti C, Blalock S, Ikemba C (2012). Hypertrophic cardiomyopathy in a neonate associated with nemaline myopathy. Congenit Heart Dis.

[7] Nakajima M, Shima Y, Kumasaka S, Kuwabara K, Migita M, Fukunaga Y (2008). An infant with congenital nemaline myopathy and hypertrophic cardiomyopathy. J Nippon Med Sch.

[8] Zawaideh C, Aste M, Cutuli O, Budaj I, Bezante GP, Brunelli C, Balbi M, Valbusa A (2014). Transient left ventricular and stomach apical ballooning syndromes: when the trigger is also a clinical emergency. Am J Emerg Med.

[9] Karner J, Keller H, Stöllberger C, Feichtinger H, Finsterer J (2008). Deficiency of mannose-binding lectin, myopathy, calcified endomyocardial fibrosis, and left ventricular noncompaction. Heart Lung.

[10] Nagata R, Kamimura D, Suzuki Y, Saito T, Toyama H, Dejima T, Inada H, Miwa Y, Uchino K, Umemura S, Shimizu M (2011). A case of nemaline myopathy with associated dilated cardiomyopathy and respiratory failure. Int Heart J.

[11] Gatayama R, Ueno K, Nakamura H, Yanagi S, Ueda H, Yamagishi H, Yasui S (2013). Nemaline myopathy with dilated cardiomyopathy in childhood. Pediatrics.

[12] Take C, Asano H, Komatsu A, Miyaji M, Iikuni K, Kowa H (2008). An autopsy case of dilated cardiomyopathy associated with congenital nemaline myopathy. Nihon Naika Gakkai Zasshi.

[13] Müller-Höcker J, Schäfer S, Mendel B, Lochmüller H, Pongratz D (2000). Nemaline cardiomyopathy in a young adult: an ultraimmunohistochemical study and review of the literature. Ultrastruct Pathol.

[14] Kim SY, Park YE, Kim HS, Lee CH, Yang DH, Kim DS (2011). Nemaline myopathy and non-fatal hypertrophic cardiomyopathy caused by a novel ACTA1 E239K mutation. J Neurol Sci.

[15] D’Amico A, Graziano C, Pacileo G, Petrini S, Nowak KJ, Boldrini R, Jacques A, Feng JJ, Porfirio B, Sewry CA, Santorelli FM, Limongelli G, Bertini E, Laing N, Marston SB (2006). Fatal hypertrophic cardiomyopathy and nemaline myopathy associated with ACTA1 K336E mutation. Neuromuscul Disord.

[16] Marston SB, Ingwall JS, Glueck SB (2002). Calcium, contractions, and tropomyosin Focus on “divergent abnormal muscle relaxation by hypertrophic cardiomyopathy and nemaline myopathy mutant tropomyosins”. Physiol Genomics.

[17] Skyllouriotis ML, Marx M, Skyllouriotis P, Bittner R, Wimmer M (1999). Nemaline myopathy and cardiomyopathy. Pediatr Neurol.

[18] Van Antwerpen CL, Gospe SM, Dentinger MP (1988). Nemaline myopathy associated with hypertrophic cardiomyopathy. Pediatr Neurol.

[19] Michele DE, Coutu P, Metzger JM (2002). Divergent abnormal muscle relaxation by hypertrophic cardiomyopathy and nemaline myopathy mutant tropomyosins. Physiol Genomics.

[20] Ishibashi-Ueda H, Imakita M, Yutani C, Takahashi S, Yazawa K, Kamiya T, Nonaka I (1990). Congenital nemaline myopathy with dilated cardiomyopathy: an autopsy study. Hum Pathol.

